# Influence of acetate containing fluid versus lactate containing fluid on acid-base status, electrolyte level, and blood lactate level in dehydrated dogs

**DOI:** 10.14202/vetworld.2021.2714-2718

**Published:** 2021-10-24

**Authors:** Annika Heitland, Ute Klein-Richers, Katrin Hartmann, René Dörfelt

**Affiliations:** 1 AniCura Tierklinik Haar GmbH, Haar, Germany; 2 Centre of Clinical Veterinary Medicine, Faculty of Veterinary Medicine, Clinic of Small Animal Medicine, Ludwig-Maximilians-University Munich, Munich, Germany.

**Keywords:** acid-base disorders, crystalloid solutions, fluid therapy, hyperlactatemia, metabolic acidosis, rehydration

## Abstract

**Background and Aim:**

Acetate or lactate buffered, balanced isotonic rehydration fluids are commonly used for fluid therapy in dogs and may influence acid-base and electrolyte status. This study aimed to assess acid-base status, electrolyte levels, and lactate levels in dehydrated dogs after receiving acetate or lactate-containing intravenous rehydration fluids.

**Materials and Methods:**

In this prospective, randomized study, 90 dehydrated dogs were included and randomized to receive acetate [Sterofundin^®^ ISO B. Braun Vet Care (STERO), Germany) or lactate (Ringer-Lactat-Lösung nach Hartmann B. Braun Vet Care (RL), Germany] containing intravenous fluids for rehydration. The exclusion criteria were as follows: Age <6 months, liver failure, congestive heart failure, and extreme electrolyte deviation. Physical examination, venous blood gas, and lactate levels were analyzed before and after rehydration. The two groups were compared using t-test and Chi-square test. The significance level was set at p≤0.05.

**Results:**

Post-rehydration heart rate decreased in the STERO group (p<0.001) but not in the RL group (p=0.090). Lactate levels decreased in both groups STERO (p<0.001) and in group RL (p=0.014). Sodium and chloride levels increased during rehydration in group STERO (p<0.001; p<0.001) and group RL (p=0.002; p<0.001). There was a larger decrease in lactate levels in group STERO compared to group RL (p=0.047).

**Conclusion:**

Both solutions led to a mild increase in sodium and chloride levels and decreased lactate levels. The acetate-containing solution had an inferior effect on the decrease in lactate level.

## Introduction

Dehydration is a common deviation in critically ill dogs. Dehydration can be caused by increased fluid loss, such as vomitus, diarrhea, and polyuria, and decreased fluid intake due to anorexia, nausea, or critical illness [1]. Therefore, the application of intravenous rehydration fluids is a cornerstone of therapy in these patients [2,3]. The appropriate amount of fluid is calculated based on the estimated dehydration, to which the expected losses and maintenance fluids are added [4].

Isotonic crystalloid fluids are commonly used for rehydration. These contain electrolytes and water, which diffuse depending on the osmolarity between the vascular and interstitial compartments [5]. The classical isotonic crystalloid, isotonic saline contains a supraphysiologic chloride concentration. The application of large amounts of isotonic saline leads to the development of hyperchloremic metabolic acidosis [6]. This may cause an increase in potassium and promote the expression of pro-inflammatory cytokines in experimental sepsis [7].

A more physiological approach is the administration of balanced isotonic crystalloid solutions. The electrolyte concentration is closer to the patient’s plasma concentration and includes potassium, calcium, and sometimes magnesium. Some balanced rehydration fluids contain buffer substances, such as acetate or lactate. Balanced isotonic rehydration fluids currently available are acetated solutions (e.g., Sterofundin^®^ ISO, B. Braun Vet Care, Germany^,1^) and lactated solutions (e.g., Ringer-Lactat-Lösung nach Hartmann, B. Braun Vet Care, Germany). Besides their buffer molecules, they also differ in electrolyte composition. Sterofundin ISO^®^ contained a higher amount of chloride and a lower amount of potassium (Table-1). The content of different buffers in rehydration fluids may influence metabolism, oxygen consumption during the metabolism of buffer substances, and acid-base status. Buffers are converted to bicarbonate and help maintain or increase the extracellular pH [8,9]. Acetate is mainly metabolized in the skeletal muscle [9]. Metabolism is not dependent on intact liver function and does not affect blood glucose or insulin concentration [10,11]. Metabolism is not oxygen-dependent and effective at low pH values [12-15].

**Table-1:** Electrolyte composition of the rehydration solution used in 90 dehydrated dogs.

Parameter	Sterofundin ISO^®^	Ringer lactate^®^
Sodium (mmol/L)	145	130.49
Chloride (mmol/L)	127	112
Potassium (mmol/L)	4	5.37
Calcium (mmol/L)	2.5	1.84
Magnesium (mmol/L)	1.0	-
Lactate (mmol/L)	-	27.84
Acetate (mmol/L)	24	
Malate (mmol/L)	5	
Osmolarity (mosmol/L)	308	277
pH	5.1-5.9	5.0-7.0

Sterofundin®: ISO B. Braun Vet Care, Germany, Ringer lactate®: Ringer-Lactat-Lösung nach Hartmann B. Braun Vet Care, Germany

Aerobic metabolism of lactate occurs mostly in the liver and to a minor degree in the kidneys and myocardial muscle and, therefore, depends on intact liver function [13,16]. Lactate degradation is oxygen dependent, and oxygen consumption increases during lactate metabolism. During hypoxic conditions, lactate metabolism is limited [12-15]. Lactate is metabolized to some extent through the Cori cycle during ATP consumption, leading to the synthesis of glucose and increased energy consumption [17]. In addition, at very low pH values (<7.1), the activity of the key enzyme for lactate metabolism, lactate dehydrogenase (LDH), decreases, which limits the buffering capacity of lactate and severe acidosis.

The choice of rehydration fluid depends on multiple factors, such as the patient’s electrolyte and acid-base status. The influence of different solutions on acid-base status, electrolytes, and blood lactate has not been widely investigated in veterinary medicine. Two studies have described the influence of different crystalloid solutions on acid-base status and electrolytes in cats with urethral obstruction [18,19]. The effects of acetate and lactate-containing solutions have not been evaluated in dehydrated dogs.

This study aimed to investigate the influence of acetate and lactate-containing fluids on acid-base status, electrolyte levels, and blood lactate levels in dehydrated dogs.

## Materials and Methods

### Ethical approval

The study protocol was approved by the ethics committee of the Center of Clinical Veterinary Medicine (number 153-09-12-2018).

### Study period and population

This prospective, randomized clinical study was conducted from April 2019 to July 2020. Ninety client-owned, dehydrated dogs presenting to the clinic due to various diseases were included in the study. Dehydration was diagnosed based on the patient’s history and physical examination. The exclusion criteria were as follows: Age <6 months, liver failure, congestive heart disease, and extreme electrolyte deviations before rehydration (sodium <130 mmol/L, >160 mmol/L, potassium <3 mmol/L, and >6 mmol/L). The dogs were randomized to receive an acetated rehydration solution (STERO) or lactated solution (RL) using an open-source program (https://www.randomizer.org). During rehydration, no other fluids, electrolytes, or vasoactive drugs were administered.

Rehydration was performed by constant rate infusion for 6-24 h, depending on the patient’s requirements. Suspected or measured ongoing loss and maintenance fluids (mL/kg/h=70×kg^0.75^/24) were administered simultaneously. Physical examination and venous blood gas analysis were performed before and after the calculation of rehydration time.

### Blood collection and laboratory analysis

Blood was collected by vein puncture or three-syringe technique from an indwelling venous catheter in the cephalic or saphenous vein before and after confirmed rehydration without exposure to room air and was analyzed within 10 min (IDEXX VetStat and IDEXX Catalyst Dx, IDEXX Laboratories, Westbrook, USA). The analysis was performed with the temperature corrected for the patient’s temperature.

### Statistical analysis

Statistical analysis was performed using commercial software (Prism 5 for Windows, GraphPad Software, Inc., San Diego, USA). Data are presented as mean±standard deviation. Age, weight, amount of fluid, electrolyte concentration, lactate, and acid-base status were analyzed using the t-test. Survival was analyzed using the Chi-square test. Statistical significance was set at p≤0.05.

## Results

The mean age and weight of the 90 dehydrated dogs were 7.4±4.5 years and 17.0±11.9 kg, respectively. Twenty-four dogs were intact females, 24 spayed females, 27 intact males, and 15 castrated males. The included breeds were mixed breed (n=20), Labrador Retriever (n=7), Yorkshire Terrier (n=7), Chihuahua (n=4), Golden Retriever (n=4), German Shepherd (n=4), and West Highland White Terrier (n=4), and other breeds represented <4 individuals per breed. The main causes of dehydration were gastrointestinal fluid loss (n=49), gastrointestinal obstruction (n=13), inflammatory diseases (n=10), neurological diseases (n=7), neoplastic diseases (n=6), infectious diseases (n=2), and others (n=3). Demographic data, amount and duration of fluid administration, and survival were not significantly different between the two groups (Table-2).

**Table-2:** Demographic data of 90 dehydrated dogs receiving either acetated (STERO) or lactated (RL) rehydration fluids.

Parameter	STERO (n=45)	RL (n=45)	p-value
Age (years)	7.6±4.4	7.1±4.7	0.595
Weight (kg)	16.5±12.6	17.4±11.4	0.707
Dehydration (%)	5.6±1.1	5.8±1.2	0.415
Amount of fluid (mL)	1668±1329	1775±1268	0.698
Amount of fluid (mL/kg)	105±23	109±30	0.525
Fluid rate (mL/h)	106±80	113±80	0.665
Duration of rehydration (hours)	16.2±5.1	17.3±4.6	0.284
Fluid rate (mL/kg/h)	7.0±2.2	6.5±1.6	0.212
Survival	41/45	39/45	0.739

Statistically significant at p≤0.05

Post-rehydration heart rate decreased significantly in the STERO group (p*<*0.001) but not in the RL group (p*=*0.090; Table-3). Lactate levels decreased during rehydration in the STERO group (p<0.001) and in the RL group (p*=*0.014; Table-3). Sodium and chloride levels increased during rehydration in the STERO (p<0.001; p<0.001) and RL (p*=*0.002; p<0.001; Table-3) groups. When comparing the differences in heart rate, acid-base status, lactate, and electrolytes, only lactate showed a stronger decrease in the STERO group than the RL group (p*=*0.047; Table-4).

**Table-3:** Vital parameters, venous blood gas values, lactate, and electrolytes before (pre) and after (post) rehydration with acetated (STERO) or lactated (RL) rehydration fluids in 90 dogs compared with t-test.

Parameter	STERO (n=45)			RL (n=45)		
	
	Pre	Post	p-value	Pre	Post	p-value
Heart rate (/min)	127±22	113±18	<0.001*	122±23	115±20	0.090
Temperature (°C)	38.3±0.7	38.2±0.6	0.497	38.4±1.0	38.4±0.8	0.874
pH	7.40±0.08	7.41±0.05	0.155	7.40±0.07	7.40±0.07	0.802
CO_2_ (kPa)	37.2±5.4	37.1±5.3	0.909	34.5±5.4	35.3±6.3	0.230
Bicarbonate (mmol/l)	21.3±4.6	21.8±3.7	0.309	19.8±3.8	19.9±3.5	0.761
Base excess	−1.6±5.0	−0.8±3.6	0.146	−2.6±3.7	−2.4±3.7	0.698
Anion gap	23.8±4.7	24.3±4.0	0.345	25.0±3.7	24.9±3.2	0.700
Lactate (mmol/l)	3.2±2.2	1.7±0.8	<0.001*	2.6±1.6	1.9±1.0	0.014*
Sodium (mmol/l)	156±5	160±4	<0.001*	156±4.9	158±4.0	0.002*
Potassium (mmol/l)	3.8±0.6	3.8±0.6	0.783	3.9±0.4	3.9±0.4	0.833
Chloride (mmol/l)	115±4	117±3	<0.001*	116±3	118±3	<0.001*

Statistically significant at p≤0.05

**Table-4:** Pre- and post-rehydration differences of vital parameters, venous blood gas values, lactate, and electrolytes before and after rehydration with acetated (STERO) or lactated (RL) rehydration fluids in 90 dogs compared with t-test.

Parameter	STERO	RL	p-value
Heart rate (/min)	−14±23	−7±27	0.196
Temperature (°C)	−0.1±0.8	0.0±1.1	0.586
pH	0.02±0.08	0±0.06	0.241
CO_2_ (kPa)	−0.2±6.5	0.8±4.5	0.389
Bicarbonate (mmol/l)	0.5±3.8	0.1±2.9	0.637
Base excess	0.6±4.1	0.2±3.5	0.594
Anion gap	0.5±7.8	−0.2±3.0	0.357
Lactate (mmol/l)	−1.5±2.3	−0.7±1.7	0.047*
Sodium (mmol/l)	4±4	2±4	0.065
Potassium (mmol/l)	0.0±0.5	0.0±0.4	0.659
Chloride (mmol/l)	2.6±2.8	1.8±3.3	0.216

Statistically significant at p≤0.05

## Discussion

Administration of intravenous fluids to dehydrated or hypovolemic animals is an important therapeutic tool in critical care medicine [3]. More than half of the dogs in this study were dehydrated due to gastrointestinal disorders (diarrhea, vomiting, and inappetence), which are commonly seen in veterinary medicine [20]. Gastrointestinal symptoms can easily cause dehydration and hypovolemia due to decreased intestinal fluid resorption and increased gastrointestinal fluid loss [20,21].

In this study, sodium and chloride levels significantly increased after rehydration in dogs in both groups. Potassium levels did not change in either group. Two previous veterinary studies also did not find an impact of isotonic balanced crystalloids containing physiological amounts of potassium on potassium levels [18,19]. In a study of 68 cats with naturally occurring urethral obstruction, randomized to receive 0.9% sodium chloride or an acetate/gluconate-containing solution, 22 were initially hyperkalemic [19]. Normalization of potassium was similar between both groups and was not affected by the chosen fluid [19]. The effect of intravenous fluid therapy with lactated Ringer’s and 0.9% sodium on renal and cardiorespiratory parameters was evaluated in 10 cats with experimentally induced urethral obstruction. Lactated Ringer’s was a superior stabilization electrolyte compared to 0.9% sodium chloride. In addition, potassium levels did not increase in the lactated Ringer’s group [18].

In this study, due to the lower sodium content, hyponatremia was expected in the RL group. In contrast, Sterofundin ISO^®^ contains a supraphysiologic amount of chloride, which may cause hyperchloremia. Following rehydration, these electrolytes did not differ between the two groups. This may be due to the long period of intravenous rehydration, during which the electrolytes could have been adjusted by renal compensation and elimination.

In this study, no statistically significant differences in post-rehydration acid-base status were observed between the two groups. In human medicine, acetate-containing solutions are suspected to cause metabolic alkalosis [22-27]. However, in 148 patients undergoing cardiac surgery receiving acetate or lactate-containing intravenous fluids, metabolic alkalosis was not observed after administration of an acetate-containing solution [28]. In 68 cats with naturally occurring urethral obstruction, the acid-base status normalized faster using the acetate/gluconate-containing solution compared to isotonic saline [19]. Acid-base status was analyzed only before and after rehydration in this study; therefore, no information on the speed of acid-base changes can be obtained. In addition, dogs with severe acid-base changes were not included in the study.

Lactate levels decreased in both groups after rehydration, but lactate reduction was stronger in the STERO group. Lactate exists in two different stereoisomers (D- and L-stereoisomers). Mammalian species metabolize lactate, which is also measured by a blood gas analyzer [29]. D-lactate is mainly produced by gastrointestinal bacteria and is used in some lactated fluids [30]. The metabolism of both stereoisomers requires stereoisomer-specific LDH. After administration of large amounts of lactated Ringer’s, blood lactate values increase in humans [31]. The acetated intravenous solution reduced the blood lactate level compared to lactated Ringer’s in 60 human patients who underwent free-flap reconstructive surgery. Blood lactate increased with longer administration of lactated Ringer’s, indicating a reduction in hepatic lactate metabolism under general anesthesia [14].

In this study, dogs aged <6 months and dogs with liver failure were excluded, as these animals were unable to adequately metabolize lactate [32]. In addition, blood lactate concentration is higher in puppies than in adult dogs [33].

The general decrease in blood lactate levels in both groups can be explained by the improvement of the underlying disease and tissue perfusion. Larger lactate level reduction in the STERO group could be explained by additional lactate intake by infusing lactated Ringer’s. The use of a racemic lactate mixture of L-lactate and D-lactate, which are used in some countries, could lead to an additional increase in blood lactate levels.

The larger decrease in heart rate in the STERO group compared to the RL group was statistically significant but clinically irrelevant. The dogs in the STERO group could have been more critically ill or could have different ongoing losses than expected. In this study, illness severity scores were not used.

A limitation of this study is the small sample size. Power analysis was performed to detect differences in sodium and chloride concentrations of 5 mmol/L. The assessment of dehydration was performed subjectively and is especially difficult in patients with cachexia or obesity, as well as in dogs with moist mucous membranes. However, dehydration was used as an indication for fluid therapy and was not a primary outcome measure.

Rehydration time was not standardized but was adapted to dehydration at the discretion of the clinician. Therefore, the individual amounts of fluids were different and also influenced by ongoing losses. A standardized fluid therapy protocol using rehydration fluids over a defined time period at a defined fluid rate would be beneficial for further experimental studies.

Dogs aged <6 years, dogs with liver disease, and dogs with congestive heart failure were excluded, as they would not have tolerated large amounts of fluids in patients with congestive heart failure. Due to the aforementioned exclusion criteria, the results of this study cannot be transferred to severely critically ill dogs, dogs with liver or congestive heart failure, or puppies.

## Conclusion

Both solutions were capable of correcting acid-base and electrolyte disturbances and reducing blood lactate levels. No solution appeared inferior.

## Authors’ Contributions

AH: Designed the study, conducted literature review, performed the study, interpreted data, and drafted manuscript. UK and KH: Designed the study and reviewed the manuscript. RD; Designed the study, interpreted data, and reviewed the manuscript. All authors read and approved the final manuscript.
